# Effectiveness of Silent Mentor Program (SMP) Workshop on Enhancing Confidence in Surgical Skills

**DOI:** 10.5704/MOJ.2503.006

**Published:** 2025-03

**Authors:** LP Wong, H Alias, SL Tan, SL Khaing, TE Sia, A Saw

**Affiliations:** 1 Centre for Population Health (CePH), Department of Social and Preventive Medicine, Universiti Malaya, Kuala Lumpur, Malaysia; 2 Fujian Key Laboratory of Environmental Factors and Cancer, Department of Epidemiology and Health Statistics, Fujian Medical University, Fuzhou, China; 3 Department of Medicine, Korea University, Seoul, Republic of Korea; 4 National Orthopaedic Centre of Excellence for Research and Learning (NOCERAL), Department of Orthopaedic Surgery, Universiti Malaya, Kuala Lumpur, Malaysia; 5 Unit of Silent Mentor Program, Universiti Malaya, Kuala Lumpur, Malaysia

**Keywords:** body donor, cadaveric dissection, chest tube insertion, endotracheal intubation, skin suturing

## Abstract

**Introduction:**

Simulation-based surgical skills workshops using ‘Silent Mentors’ are employed in numerous surgical training programs worldwide, yet empirical evidence on their effectiveness remains limited. The objective of this study was to investigate whether participation in the surgical skills workshop within the Silent Mentor Program (SMP) resulted in an improvement in the surgical skills of the workshop attendees.

**Material and Methods:**

Participants in the SMP at Universiti Malaya during the period from May 15, 2022, to September 24, 2023, were included in the study. Participants self-evaluated their surgical skill confidence levels in four fundamental surgical skills (chest tube insertion, central venous line insertion, endotracheal intubation, and skin suturing). The pre-workshop confidence scores were assessed and compared with immediate post-workshop scores.

**Results:**

The findings demonstrated that after the training, participants exhibited higher confidence in all four fundamental surgical skills. Skin suturing demonstrated the highest total confidence score post-workshop, with a median of 21 and an interquartile range (IQR) of 18-24. Endotracheal intubation and chest tube insertion followed closely, both with a median of 19. Conversely, central line insertion displayed the lowest total confidence score, registering a median of 18 (IQR=16-21). No statistically significant differences were observed in the confidence level scores for chest tube insertion, central line insertion, and endotracheal intubation between pre- and post-workshop assessments across all demographic characteristics.

**Conclusion:**

In conclusion, utilizing silent mentors in surgical skills training enhances proficiency in all four fundamental surgical skills, with skin suturing demonstrating particularly noteworthy improvements. The consistent confidence levels across demographic factors suggest the workshop's effectiveness across a broad spectrum of participants.

## Introduction

The Silent Mentor Program (SMP) represents a unique educational model that integrates surgical skill training while instilling humanistic values in simulation-based surgery teaching^1^. It is founded on the noble principle of body donation, turning the act of donating one's body into a meaningful gift. In contrast to the traditional cadaver donation, where the cadaver remains unidentified, the SMP forefronts the identity of the training cadaver as an essential element in medical pedagogy, deliberately engaging the student with the family of the deceased and aiming to build long relationships between students and their mentors^2^. Most of the donors in the SMP would have registered as pledgers long before they were ill. The next-of-kin of the pledger was made known to contact the centre when the pledger is critically ill. Upon the death of the pledger, the bodies will be kept frozen at temperatures ranging from −17 to −30°C until the start of the training session to ensure that the condition of the body closely resembles that of a real person during the surgical training, hence provides a valuable opportunity for trainees to refine their surgical skills through a real-world experience with human anatomy^3^. Moving beyond traditional cadaver donation, the program serves a dual purpose by providing surgical skill training, emphasizing humanity training, for instance respect and gratitude for the donors, and fostering altruism in students^4^.

Acquiring fundamental surgical skills forms a cornerstone of medical education, and among these essential skills are chest tube insertion, central venous line insertion, endotracheal intubation, and skin suturing. Recognised as integral components of surgical training, these procedures form a crucial part of SMP training at the Universiti Malaya^1^. The Universiti Malaya in Malaysia introduced the SMP in the year 2012. For undergraduate medical students, the training encompasses these four essential procedures. This program, delivered through a tightly scheduled four-day surgical training series, also provides advanced surgical skill training for surgeons and clinical specialists. The advanced training includes specialized courses in laparo-endoscopic surgery, plastic surgery, arthroscopy, arthroplasty, trauma surgery, limb deformity correction, ophthalmology, and oral and maxillofacial procedures.

While the SMP in Universiti Malaya has incorporated training in these fundamental surgical skills for undergraduate medical students since its establishment, there is a scarcity of evidence regarding on the effectiveness of surgical training using human bodies. Assessing the effectiveness of such training is crucial for ensuring that medical graduates acquire the necessary skills and competence to perform these procedures safely and proficiently in real-life clinical settings. Additionally, a comprehensive evaluation can also inform program improvements and contribute to the overall enhancement of SMP surgical skill training.

Therefore, this study aimed to explore if the four-day surgical skills workshop in the SMP enhanced the skills of workshop participants. To evaluate surgical training effectiveness, we will study pre-workshop confidence level scores compared with post-workshop scores.

## Materials and Methods

The participants in the Silent Mentor Program (SMP), Universiti Malaya between 15th May 2022 to 24th September 2023 participated in the study. Workshop participants evaluated their own surgical skill confidence levels in performing four key procedures: chest tube insertion, central venous line insertion, endotracheal intubation, and skin suturing.

A study was conducted 15th May 2022 to 24th September 2023. All SMP participants during the program were invited to take part. This study was a quasi-experimental intervention with before and after design. Students from this University who were in their final year and those who recently graduated were invited to participate in the study. None of them had the opportunity to perform the listed procedures on living patients or cadaveric models prior to study. The trainers consisted of general surgeons, orthopaedic surgeons, anaesthetists, and surgeons from the emergency department. Most of them have volunteered to serve as trainers for more than three years. Groups of four to five students will be taught by one trainer, and they will be supervised to perform the procedures. Anticipating the workshop would increase the average score for confidence score to approximately 2.0 post-training, with a paired standard deviation of about 1, to achieve 95% power with α = 0.05, we estimated the sample size of 84 to detect medium effect size differences between the two dependent groups.

After obtaining informed consent, participants in the SMP workshop underwent assessment for demographic information, including age, gender, ethnicity, average household income, current year of study, and self-perceived academic performance. Before the workshop began, participants were given an assessment questionnaire to evaluate their skills. After the three-day workshop, the research staff distributed the same questionnaire among the participants to assess their perceived confidence in performing the surgical skills they had learned. The pre-workshop confidence scores of participants were assessed and compared with immediate post-workshop scores.

Due to the absence of an existing scale for evaluating the four fundamental surgical skills, we developed a custom assessment scale. The assessment scale was crafted by the researcher in accordance with the learning objectives, and its content was subsequently verified through validation by a panel of experts. The surgical skills assessment is shown in Appendix 1. The questionnaire is designed to evaluate participants' confidence in three key areas: identifying the surface landmark, familiarity with the anatomy, managing potential complications, prescribe post- procedure management, and performing the procedure under and without supervision. A four-point Likert scale (with item scores ranging from 1 to 4) was employed to evaluate participants' self-assessed confidence regarding their performance both before and after the workshop. The response options include extremely, somewhat, little, and not at all, corresponding to numerical ratings of 1 to 4, respectively. Total scores were calculated, with higher scores indicating a greater level of confidence. Each surgical skill had six question items, resulting in a score range of 4 to 24 for each skill.

Descriptive statistics for the demographic characteristic variables and confidence levels were presented using frequencies, percentages, median, and interquartile range (IQR). Pre- and post-workshop mean confidence scores for each surgical skill were calculated and stratified across demographic factors. Paired t-tests and analysis of variance (ANOVA) were employed to test hypotheses, comparing means within and between groups, respectively. The Student's t-test is applied to compare means between two groups, while ANOVA is used to assess means across three or more groups. Descriptive statistics and statistical analysis were conducted using SPSS Statistics for Windows [IBM Corporation, Armonk, USA].

This study was implemented according to the principles of the Declaration of Helsinki and granted ethical approval by the Universiti Malaya Research Ethics Committee (UM.TNC2/UMREC–1210). Study participants were also informed that their participation was voluntary. Informed consent was acquired, and participants were explicitly instructed to provide honest opinions.

## Results

A total of 211 participants completed both pre- and post-surgical skills assessments. The participants' ages ranged from 22 to 26 years old. Among them, 61.6% (n=130) were female, while 38.4% (n=81) were male. Most participants were Chinese (58.8%), and a significant number came from families with an average monthly income exceeding MYR8,000 (USD1,700). In terms of academic status, 58.3% were final-year students, and a smaller percentage were recent graduates (11.4%). Regarding self-reported academic performance, approximately half of the participants considered their performance as poor or fair (48.3%), while the other half rated it as good or excellent (51.7%).

**Fig. 1:**
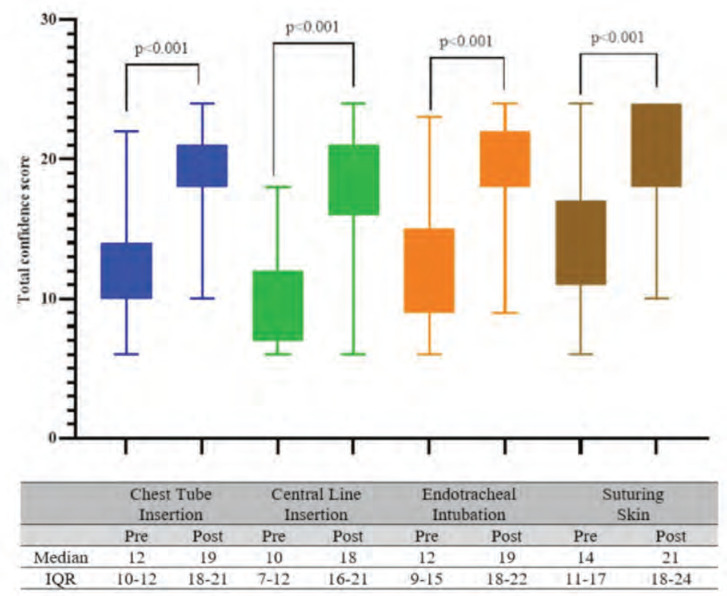
Comparison of the total confidence score for the four fundamental surgical skills pre- and post-workshop (N=211)

As depicted in Fig. 1, the comparison of the total confidence scores for the four fundamental surgical skills revealed a significant increase in the total confidence score from pre- to post-workshop in the four fundamental surgical skills. The suturing of skin exhibited the highest post-workshop total confidence score, with a median of 21 (IQR= 18-24). In contrast, the lowest post-workshop total confidence score was observed for central line insertion, with a median of 18 (IQR =16-21). Endotracheal intubation (median=19, IQR=18-22) and chest tube insertion (median 19, IQR= 1821) recorded near similar post-workshop confidence scores. Table I shows the confidence levels of items in pre- and post-workshop assessments for the four fundamental surgical skills. For skin suturing, the median confidence scores pre-workshop were 2 and 3 for all items. In the post-workshop assessment, three surgical skills (familiarity with different methods in skin closure, understanding various layers of skin and subcutaneous tissue, and performing the procedure under supervision) achieved high median scores (median = 4, IQR = 3-4 confidence scores).

**Table I:** Confidence levels of items in pre- and post-workshop assessments for the four fundamental surgical skills.

Surgical skill training	Level of confidence Median (IQR)		
	Pre	Post	p-value
**Chest Tube Insertion**			
Identify the landmarks for skin incision	2 (2-3)	4 (3-4)	p<0.001
Familiar with the anatomy of the region	3 (2-3)	4 (3-4)	p<0.001
Manage potential complication	1 (1-2)	3 (2-3)	p<0.001
Prescribe post procedure management	2 (1-2)	3 (3-3)	p<0.001
Performing the procedure under supervision	2 (2-3)	4 (3-4)	p<0.001
Performing the procedure without supervision	1 (1-2)	3 (2-3)	p<0.001
**Central Line Insertion**			
Identify the landmark for line insertion	2 (1-2)	3 (3-4)	p<0.001
Familiar with the anatomy of the region	2 (1-3)	3 (3-4)	p<0.001
Manage potential complication	1 (1-2)	3 (2-3)	p<0.001
Prescribe post procedure management	1 (1-2)	3 (2-3)	p<0.001
Performing the procedure under supervision	2 (1-3)	3 (3-4)	p<0.001
Performing the procedure without supervision	1 (1-2)	3 (2-3)	p<0.001
**Endotracheal Intubation**			
Familiar with the instruments used for this procedure	3 (2-3)	4 (3-4)	p<0.001
Familiar with the anatomy of the region	2 (2-3)	3 (2-3)	p<0.001
Manage potential complication	2 (1-2)	3 (2-3)	p<0.001
Prescribe post procedure management	2 (1-2)	3 (3-4)	p<0.001
Performing the procedure under supervision	2 (2-3)	4 (3-4)	p<0.001
Performing the procedure without supervision	1 (1-2)	3 (2-3)	p<0.001
**Suturing Skin**			
Familiar with different methods in skin closure	2 (2-3)	4 (3-4)	p<0.001
Familiar with various layers of skin and subcutaneous tissue	3 (2-3)	4 (3-4)	p<0.001
Manage potential complication	2 (1-3)	3 (3-4)	p<0.001
Prescribe post procedure management	2 (1-3)	3 (3-4)	p<0.001
Performing the procedure under supervision	3 (2-3)	4 (3-4)	p<0.001
Performing the procedure without supervision	2 (1-3)	3 (3-4)	p<0.001

In chest tube insertion training, post-workshop median scores of 4 (IQR = 2-3) were reported for identifying surface landmarks for skin incision, familiarity with the anatomy of the region, and performing the procedure under supervision. In the pre-workshop assessment, the median confidence score for managing potential complications and performing procedures without supervision was the lowest (median = 1, IQR = 1-2). However, in the post-workshop assessment, the median score increased to 3 (IQR = 2-3) with a significant difference (P < 0.001).

Regarding endotracheal intubation training, the median confidence score was lowest in performing the procedure without supervision (median = 1, IQR = 1-2) in the pre-workshop assessment and increased to 3 (IQR = 2-3) in post-workshop evaluation. A median confidence score of 4 (IQR = 3-4) was reported for being familiar with the instruments used for this procedure and performing procedures without supervision in the post-workshop.

For central line insertion, the median confidence score for managing potential complications and performing procedures without supervision was the lowest (median = 1, IQR = 1-2) in the pre-workshop assessment. In the post-workshop assessment, a median confidence score of 3 was reported for all items in central line insertion training.

The comparison of surgical skills confidence levels pre- and post-workshop by background characteristics is shown in Table II. No statistically significant difference was observed in the confidence level scores for chest tube insertion, central line insertion, and endotracheal intubation between pre- and post-workshop assessments across all demographic characteristics. Similarly, there were no statistically significant differences in the confidence level scores for skin suturing between pre- and post-workshop assessments across all demographic characteristics, except for gender and ethnicity. Although both genders demonstrated comparable confidence scores for skin suturing post-workshop (total score = 21, IQR 18-24), significant differences were observed between pre- and post-workshop confidence scores based on gender. The female participants exhibited a more substantial elevation in skin suturing confidence level scores, with an increase from 14 (9-16.3) to 21 (18-24). Regarding ethnicity, Malays and individuals from other ethnic backgrounds exhibited the highest post-workshop confidence scores for skin suturing.

**Table II:** Comparison of total surgical skills confidence score pre-and post-workshop by background characteristics (N=211).

Socio demographic characteristics	N (%)	Chest Tube Insertion			Central Line Insertion			Endotracheal Intubation			Suturing Skin		
		Pre	Post	p-value	Pre	Post	p-value	Pre	Post	p-value	Pre	Post	p-value
Age group (years)		Median	Median		Median	Median		Median	Median		Median	Median	
		(IQR)	(IQR)		(IQR)	(IQR)		(IQR)	(IQR)		(IQR)	(IQR)	
22-23	78 (37.0)	12 (10-14)	20 (18-22)	0.807	11 (7-12.3)	18 (17-21)	0.678	12 (10-15)	20.5 (18-22)	0.676	15 (12-18)	22 (19-24)	0.872
24-26	133 (63.0)	12 (9-14)	19 (17.5-21)		10 (6.5-12)	18 (15-20)		12 (8-15)	19 (18-22)		14 (10-17)	21 (18-24)	
**Gender**													
Male	81 (38.4)	12 (10-14.5)	19 (18-22)	0.082	11 (7-12)	18 (16-21)	0.562	13 (11-15.5)	21 (18-23)	0.237	15 (12-18)	21 (18-24)	0.003
Female	130 (61.6)	12 (9-14)	19 (18-21)		10 (7-12)	18 (16-21)		12(8-14)	19 (18-22)		14(9-16.3)	21 (18-24)	
**Ethnicity**													
Malay	50 (23.7)	12.5 (10.8-14)	20 (18-21.3)	0.798	11 (6.8-12.3)	18.5 (15-21)	0.302	13 (9-14)	21 (18-22)	0.183	14 (11-17)	22 (20-24)	0.001
Chinese	124 (58.8)	12 (9-14)	19 (17-21)		9.5 (6.3-12)	18 (16-21)		12 (8-14.5)	19 (18-22)		13 (9-17)	21 (18-24)	
Indian	26 (12.3)	12 (10.8-15)	21 (18-22)		12 (9.8-14)	18 (16.8-21)		12 (11.8-17)	18.5 (17-22.3)		16.5 (12.8-18)	20(18-24)	
Others	11 (5.2)	10 (8-16)	20 (18-21)		9(7-15)	18 (18-21)		11 (7-16)	19 (18-22)		14 (12-15)	22 (18-24)	
**Average monthly household Income (MYR)**													
<4000	59 (28.0)	12 (9-14)	19 (17-21)	0.642	10 (7-13)	18 (15-21)	0.515	12 (9-15)	19 (18-23)	0.833	14 (10-18)	21 (18-24)	0.919
4001-6000	39 (18.5)	12 (10-15)	20 (18-21)		10 (8-13)	18 (17-21)		12 (8-15)	20 (18-22)		13 (10-17)	21 (18-23)	
6001-8000	36 (17.1)	11.5 (10-13.8)	18.5 (17-21)		10(6-12.8)	18 (17-21.5)		12 (8-14.8)	19.5 (17.3-22)		13.5 (9.3-16)	21.5 (18-24)	
>8000	77 (36.5)	12 (10-14)	20 (18-21.5)		10 (7- 12)	18 (15-21)		12 (9-14.5)	19 (18-22)		15 (11.5-17.5)	21 (18.5-24)	
**Education background Study year**													
3rd/4th year	64 (30.3)	12 (10-14)	19 (18-21)	0.848	11 (8-13)	18 (17-21)	0.637	12.5 (10-16)	20 (18-22)	0.361	15 (12-18)	22 (19.3-24)	0.321
Final year	123 (58.3)	12 (10-14)	19 (18-22)		11 (7-12)	18 (15-21)		12 (9-15)	19 (18-22)		14 (11-17)	21 (18-24)	
Graduated	24(11.4)	10 (8-13.8)	18.5 (17-20.8)		7.5 (6-11)	17 (15-19)		10.5 (6.3-13.8)	19 (17-21)		12.5 (8-14.8)	20 (18-22.8)	
**Self-rating of academic performance**													
Poor/Fair	102 (48.3)	12 (9-14)	19 (17-21)	0.816	10 (6-12)	18 (16-21)	0.539	12 (8-15)	19 (17.8-22)	0.696	14 (10.8-16)	21 (18-24)	0.967
Good/Excellent	109 (51.7)	12 (10-14)	20 (18-21.5)		11 (7-13)	18 (16-21)		12 (9-15)	20 (18-22)		14 (11-17.5)	21 (19-24)	

## Discussion

The findings presented a notable increase in total confidence scores for the four fundamental surgical skills from pre- to post-workshop. This significant improvement suggests that the workshop effectively enhanced participants' overall confidence levels in these critical surgical skills. The findings of the confidence levels of individual items in pre-and post-workshop assessments for the four fundamental surgical skills provide a detailed perspective on the workshop's effectiveness in bolstering participants' confidence across diverse skills.

Skin suturing has been the most popular surgical skill training sessions among medical students^5^. Cadaveric skin and animal skin are generally considered high fidelity models while biosimilar silicon and other synthetic materials provide alternative options at lower cost^6^. In our study, participants exhibited the highest confidence levels in skin suturing. In the domain of skin suturing, three specific skills (namely familiar with different methods in skin closure, understanding various layers of skin and subcutaneous tissue, and performing the procedure under supervision) achieved heightened levels of confidence post-workshop. Wound management stands as a crucial aspect within the realm of emergency medicine practice. Clinicians are tasked with attending to a spectrum of wounds, encompassing both minor and uncomplicated lacerations or abrasions to intricate and challenging wound cases. An understanding of the fundamental characteristics of suture materials and surgical needles is necessary if one is to obtain optimal surgical results and treatment outcome. The proficiency gained in the workshop, including familiarity with diverse skin closure techniques and a comprehensive understanding of the various layers of skin and subcutaneous tissue, equips participants with valuable skills. These acquired skills are instrumental in enhancing participants' capabilities in addressing a broad array of wounds, ensuring effective and adept wound management in emergency medical scenarios.

In the context of chest tube insertion, three out of the six assessed surgical skills (namely identifying landmarks for skin incision, familiarity with regional anatomy, and performing the procedure under supervision) attained the highest levels of confidence post-workshop. This highlights the workshop's success in enhancing participants' proficiency and assurance in these critical aspects of chest tube insertion. The insertion of a chest tube is a common intervention for managing various thoracic injuries, nevertheless, it is linked to the potential for severe complications^7^. This perhaps explains the low pre-workshop scores in performing the procedure without supervision and managing potential complications, as it involves penetrating the thoracic cage in the vicinity of the heart and major vessels. Multiple studies have highlighted the necessity for a structured training program to mitigate the complications associated with chest tube insertion and improve overall patient safety^8^. In the current workshop participants reported gaining high level of confidence in identifying landmarks for skin incision and familiarity with the anatomy of the region. It is important to highlight that the participants in the SMP workshop achieved heightened confidence in these skills. This underscores the significant benefits of the SMP workshop for enhancing proficiency in chest tube insertion among the workshop attendees. In future chest tube insertion training sessions, integrating the domains of "preprocedural checks" and "patient positioning and local anaesthetic," as recommended by Kuper *et al*^9^, may potentially improve resident performance and ensure patient safety.

Like chest tube insertion, participants' confidence in performing endotracheal intubation post-workshop was somewhat lower compared to suturing skin. However, we observed a two-point improvement in confidence levels in performing the procedure with and without supervision when comparing levels before and after the workshop. Securing the airway is the first step during the resuscitation of sick or injured patients, and there is hardly any time to ask for assistance^10^, thus this training method proves to be very useful. According to a recent study, emergency trainees needed a minimum of 119 attempts at endotracheal intubation to achieve a first attempt success rate of more than 85% in the emergency department^11^. In addition, a study conducted in the United States indicated that 40% of pulmonary and critical care medicine trainees expressed concerns about their proficiency in endotracheal intubation upon graduation^12^. This implies that endotracheal intubation training in SMP served as an essential platform and crucial foundation for skills development for medical students or graduates.

Central line insertion exhibited the lowest confidence level post-workshop compared to other surgical skills, with none of the items under this skill achieving heightened levels in the post-workshop assessment. A median confidence score of 3, out of the maximum 4 rating, was reported for all items in central line insertion training. A retrospective study, based on students’ logbooks showed that the satisfaction and confidence of medical students regarding their performance in central line insertion increased with each additional procedure and decreased significantly if failure or complications had occurred^13^. In view of the challenges faced by medical students, such as the difficulty of finding the central veins and puncturing arteries^13^, the SMP training session can provide an opportunity to practice this specific skill, thereby addressing the major challenge of locating central veins. However, without arterial pulsation and venous backflow, it may be difficult for the trainer and participants to be sure of successful cannulation during central line insertion, which explains the lowest confidence level post-workshop compared to other surgical skills. This outcome aligns with expectations, considering that maintaining the circulation is the second most important aspect of resuscitation following establishing a patent airway^14^, particularly in cases of patients who have collapsed due to hypovolaemic shock or severe dehydration. To address this, there may be a need to improve or modify the current teaching protocol, or even consider a different approach for training in this procedure.

Table II further explores the impact of background characteristics on surgical skills confidence levels, both before and after the workshop. Notably, no statistically significant differences were detected in confidence scores for chest tube insertion, central line insertion, and endotracheal intubation when comparing pre-and post-workshop assessments across various demographic characteristics. This uniformity suggests that the workshop's positive influence on confidence levels was consistent across diverse participant backgrounds. In the context of skin suturing, the situation differed slightly. While no significant differences were found in confidence level scores across demographic characteristics in the pre-and post-workshop assessments, exceptions were noted for gender and ethnicity. While we would anticipate females to be more acquainted with needle handling, suturing, and knot tying, it is somewhat unexpected that our female participants demonstrated slightly lower pre-workshop confidence scores in skin suturing. Despite this observation, the workshop training had an equal impact on both genders, resulting in female participants attaining a level of skin suturing confidence similar to their male counterparts. Similarly, in regard to ethnic disparities, the highest post-workshop confidence scores for skin suturing were observed among Malays and individuals from other ethnic backgrounds. These differences highlight the potential influence of gender and ethnicity in shaping the response to the workshop, particularly concerning skin suturing confidence levels. Further studies are needed to explore and understand the factors contributing to these variations. Understanding these variations is crucial for tailoring future workshops or interventions to address specific needs within different demographic groups.

Despite the valuable contributions of this study, there are several limitations that warrant consideration. Firstly, participant responses are susceptible to social desirability bias, particularly reporting bias towards overestimating the effectiveness of an intervention in relation to participants' confidence levels. Secondly, self-assessed confidence may not align accurately with actual competency^15^. Evaluating actual competency in future assessments could offer a more precise measure of the workshop's efficacy. Currently many medical schools are relying on synthetic cadaveric models or manikins to teach clinical procedures, and it could be beneficial to compare these approaches with training facilitated by our mentors. Skin suturing is a simple procedure that can be performed on various skin models, and there may be other synthetic tissue models available for practicing central line cannulation. Unfortunately, we were unable to find any studies reporting their training efficiencies for meaningful comparison. Hence, the findings of this study should be interpreted in consideration of the mentioned limitations.

## Conclusions

Overall, participants in the SMP workshop expressed heightened confidence levels in each of the four fundamental surgical skills. Notably, they identified skin suturing as the skill for which they reported the highest confidence. The central line insertion exhibited the lowest total confidence score post-workshop due to training limitations. Therefore, we would consider modification of the training protocol or explore alternative training methods. The absence of significant differences in confidence scores across demographic characteristics underscores the workshop's universal benefit to participants irrespective of their diverse backgrounds. Therefore, freeze preserved human body provides an invaluable chance for medical professionals to enhance their surgical expertise by immersing themselves in a real human anatomy experience. Simultaneously, it may also foster a profound sense of empathy and humanistic values in healthcare practitioners.

## Appendix

### Appendix 1: Questionnaire

**SILENT MENTOR PROGRAM (SMP) SURGICAL SKILL ASSESSMENT**

**Table:** SECTION A : GENERAL INFORMATION

1	Age (years)	_______ years old
2	Gender	[ ] Male
		[ ] Female
3	Ethnicity	[ ] Malay
		[ ] Chinese
		[ ] Indian
		[ ] Others, please specify _______________
4	Monthly	average household income of parents (MYR) [ ] <4000
		[ ] 4001-6000
		[ ] 6001-8000
		[ ] >8000

**Table:** SECTION B: EDUCATION BACKGROUND

1	Years of study	[ ] 3rd or 4th
		[ ] Final year
		[ ] Graduated
2	Self-rating of overall academic performance	[ ] Poor
		[ ] Fair
		[ ] Good
		[ ] Excellent

**Table:** SECTION C: ASSESSMENT OF SURGICAL SKILLS

		Not at all confident	Little confident	Somewhat confident	Extremely confident
**A.**	**Chest Tube Insertion**				
1.	Identify the landmarks for skin incision	[ 1 ]	[ 2 ]	[ 3 ]	[ 4 ]
2.	Familiar with the anatomy of the region	[ 1 ]	[ 2 ]	[ 3 ]	[ 4 ]
3.	Manage potential complication	[ 1 ]	[ 2 ]	[ 3 ]	[ 4 ]
4.	Prescribe post procedure management	[ 1 ]	[ 2 ]	[ 3 ]	[ 4 ]
5.	Performing the procedure under supervision	[ 1 ]	[ 2 ]	[ 3 ]	[ 4 ]
6.	Performing the procedure without supervision	[ 1 ]	[ 2 ]	[ 3 ]	[ 4 ]
**B.**	**Central Line Insertion**				
1.	Identify the landmark for line insertion	[ 1 ]	[ 2 ]	[ 3 ]	[ 4 ]
2.	Familiar with the anatomy of the region	[ 1 ]	[ 2 ]	[ 3 ]	[ 4 ]
3.	Manage potential complication	[ 1 ]	[ 2 ]	[ 3 ]	[ 4 ]
4.	Prescribe post procedure management	[ 1 ]	[ 2 ]	[ 3 ]	[ 4 ]
5.	Performing the procedure under supervision	[ 1 ]	[ 2 ]	[ 3 ]	[ 4 ]
6.	Performing the procedure without supervision	[ 1 ]	[ 2 ]	[ 3 ]	[ 4 ]
C.	Endotracheal Intubation				
1.	Familiar with the instruments used for this procedure	[ 1 ]	[ 2 ]	[ 3 ]	[ 4 ]
2.	Familiar with the anatomy of the region	[ 1 ]	[ 2 ]	[ 3 ]	[ 4 ]
3.	Manage potential complication	[ 1 ]	[ 2 ]	[ 3 ]	[ 4 ]
4.	Prescribe post procedure management	[ 1 ]	[ 2 ]	[ 3 ]	[ 4 ]
5.	Performing the procedure under supervision	[ 1 ]	[ 2 ]	[ 3 ]	[ 4 ]
6.	Performing the procedure without supervision	[ 1 ]	[ 2 ]	[ 3 ]	[ 4 ]
**D.**	**Suturing Skin**				
1.	Familiar with different methods in skin closure	[ 1 ]	[ 2 ]	[ 3 ]	[ 4 ]
2.	Familiar with various layers of skin and subcutaneous tissue	[ 1 ]	[ 2 ]	[ 3 ]	[ 4 ]
3.	Manage potential complication	[ 1 ]	[ 2 ]	[ 3 ]	[ 4 ]
4.	Prescribe post procedure management	[ 1 ]	[ 2 ]	[ 3 ]	[ 4 ]
5.	Performing the procedure under supervision	[ 1 ]	[ 2 ]	[ 3 ]	[ 4 ]
6.	Performing the procedure without supervision	[ 1 ]	[ 2 ]	[ 3 ]	[ 4 ]
